# A mitochondrion-targeted dual-site fluorescent probe for the discriminative detection of SO_3_^2−^ and HSO_3_^−^ in living HepG-2 cells†

**DOI:** 10.1039/d0ra01233e

**Published:** 2020-07-14

**Authors:** Zhenmei Deng, Fangzhao Li, Guomin Zhao, Wenge Yang, Yonghong Hu

**Affiliations:** College of Biotechnology and Pharmaceutical Engineering, Nanjing Tech University No. 30, South Puzhu Road Nanjing 211816 China wengeyang11@163.com +86-25-58139393 +86-25-58139393

## Abstract

Sulfur dioxide, known as an environmental pollutant, produced during industrial productions is also a common food additive that is permitted worldwide. In living organisms, sulfur dioxide forms hydrates of sulfite (SO_2_·H_2_O), bisulfite (HSO_3_^−^) and sulfite (SO_3_^2−^) under physiological pH conditions; these three exist in a dynamic balance and play a role in maintaining redox balance, further participating in a wide range of physiological and pathological processes. On the basis of the differences in nucleophilicity between SO_3_^2−^ and HSO_3_^−^, for the first time, we built a mitochondrion-targeted dual-site fluorescent probe (Mito-CDTH-CHO) based on benzopyran for the highly specific detection of SO_3_^2−^ and HSO_3_^−^ with two diverse emission channels. Mito-CDTH-CHO can discriminatively respond to the levels of HSO_3_^−^ and SO_3_^2−^. Besides, its advantages of low cytotoxicity, superior biocompatibility and excellent mitochondrial enrichment ability contribute to the detection and observation of the distribution of sulfur dioxide derivatives in living organisms as well as allowing further studies on the physiological functions of sulfur dioxide.

## Abstract 2

Rational design and sensing mechanism of a dual-site fluorescence probe for HSO_3_^−^ and SO_3_^2−^.

## Graphical abstract

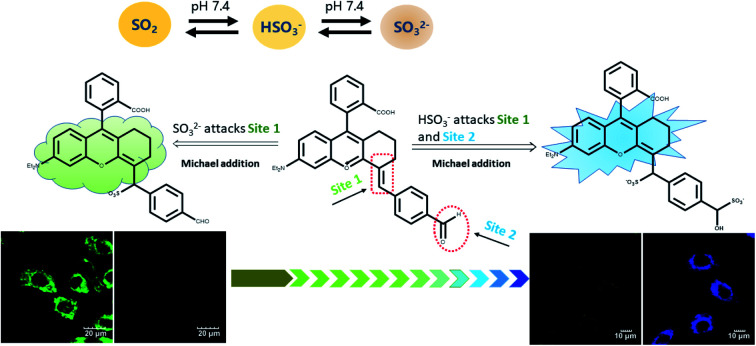

## 1. Introduction

Sulfur dioxide (SO_2_), the most common and simple irritating gas, is one of the main pollutants in the atmosphere.^1^ In recent years, increasing physiological functions of sulfur dioxide have been discovered in mammals.^2–4^ How does SO_2_ work in the internal environment of living organisms? It has been reported in numerous studies that SO_2_ is not independent in action or directly affects, but dissociated to SO_3_^2−^ and HSO_3_^−^ (SO_2_ derivatives) in neutral fluid or plasma (HSO_3_^−^/SO_3_^2−^, 1 : 3 M/M),^5,6^ which mainly account for its toxicity. There is a dynamic conversion equilibrium between sulfur dioxide and sulfite, which also exists in bisulfite and sulfite.^7^

Among them, HSO_3_^−^/SO_3_^2−^ at high concentrations are catalyzed to generate a variety of sulfur oxy radicals, which are known to give rise to negligible damage to the body.^8^ Numerous studies have confirmed that abnormally high sulfite levels are closely related to respiratory diseases,^9^ cardiovascular diseases^10^ as well as neurological diseases, such as migraine, stroke, brain cancer,^11^ lung cancer^12^ and liver cancer.^13^ Besides, clinical studies suggest that the concentration of sulfur dioxide gas ranges from 1 to 2000 μM in living organisms, and the total concentration of serum sulfite ranges from 0 to 10 μM in healthy donors. HSO_3_^−^/SO_3_^2−^ also relax aortic rings in a dose-dependent manner at high concentrations ranging from 0.5 to 12 mM.^10^ However, whether HSO_3_^−^and SO_3_^2−^ are independent or synergistic in action remains largely unknown. Therefore, the accurate and independent determination of the levels of SO_3_^2−^ and HSO_3_^−^ is fairly necessary and valuable for further investigating the physiological functions of sulfur dioxide in living organisms.

Over the past decades, fluorescence imaging technology has drawn considerable attention benefiting from its outstanding performances, such as eminent non-invasiveness, excellent signal-to-noise ratio, high sensitivity, extraordinary reliability, cheap availability and easy operation.^14^ Since Qian and Zhang *et al.* first reported a fluorescent probe based on the Michael addition reaction for HSO_3_^−^ in 2013,^15^ a large number of fluorescent probes have been developed to detect HSO_3_^−^/SO_3_^2−^ in recent years.^16–20^ Although they work on a simple mechanism and are facile to synthesize, they cannot exactly distinguish between HSO_3_^−^ and SO_3_^2−^, thus producing the same fluorescence signal. Herein, a dual-site fluorescent probe for SO_2_ derivatives (HSO_3_^−^ and SO_3_^2−^) based on benzopyran, Mito-CDTH-CHO, was designed and synthesized. The probe is capable of discriminatively responding to the levels of HSO_3_^−^ and SO_3_^2−^ with distinct fluorescence signals. Furthermore, Mito-CDTH-CHO exhibited superior selectivity, lower cytotoxicity, good sensitivity, and readily available for fluorescence imaging *in vitro* and *in vivo*. To the best of our knowledge, the probes that possess obvious fluorescence sensing for HSO_3_^−^ and SO_3_^2−^ are still rare.

## 2. Experimental section

### 2.1. Materials and instruments

All the reagents were supplied by commercial suppliers and were directly used without further purification. Absorption spectra were recorded on a UNICO UV-4802 spectrophotometer. Fluorescence spectra were obtained on a fluorescence spectrophotometer (Lengguang tech CO., Ltd. F97XP, China). ^1^H NMR and ^13^C NMR spectra were recorded on a Bruker AVANCE III 400 Nanobay at 500 MHz for ^1^H NMR and 300 MHz for ^13^C NMR (TMS as an internal standard). High-resolution mass spectra (HRMS) were recorded on a MicrOTOF Bruker. The pH values were measured with an acidity meter (alkalis, pH 400, China).

### 2.2. Synthesis of probe Mito-CDTH-CHO

The probe was synthesized according to the reported literature *via* a facile two-step reaction.^21,22^ The synthesis routes of the probe are depicted in Scheme S1,† and it was characterized *via* high-resolution mass spectrometry, ^1^H NMR and ^13^C NMR (see ESI†).

#### Synthesis of Mito-CDTH

Freshly distilled cyclohexanone was added dropwise to a solution of concentrated H_2_SO_4_ cooled down to 0 °C in advance. To a concentrated H_2_SO_4_ solution of 2-(4-diethylamino-2-hydroxybenzoyl), benzoic acid was added dropwise in freshly distilled cyclohexanone at 0 °C. Further, heating up to 90 °C, the mixture was vigorously stirred for 2 h, poured into ice, the perchloric acid (70%) was added, the supernatant was filtered off, and the residue was washed with cold water for three times. The residue was dried under vacuum and further purified *via* silica gel column chromatography (CH_2_Cl_2_ : MeOH = 20 : 1, v/v) to afford Mito-CDTH as a bright red solid (372 mg, 68%). ESI-MS calcd for C_24_H_26_NO_3_ [M + H]^+^ 376.1921, found 376.1913.

#### Synthesis of Mito-CDTH-CHO

To an acetic acid solution (30 ml) of Mito-CDTH (376 mg, 1 mmol), terephthalaldehyde (268 mg, 2 mmol) was added. The reaction solution was stirred at 110 °C for 3 h, and the solvent was evaporated under a reduced pressure. The crude product was extracted with CH_2_Cl_2_ (100 ml) and water (300 ml), washed with a saturated ammonium chloride solution and dried over anhydrous sodium sulfate. After evaporating using a rotary evaporator, the purple target compound (300 mg, 61%) was obtained *via* silica gel column chromatography (CH_2_Cl_2_ : MeOH = 50 : 1, v/v).

^1^H NMR (300 MHz, DMSO-*d*_6_) *δ* 10.03 (s, 1H), 7.95 (d, *J* = 7.8 Hz, 3H), 7.86–7.63 (m, 4H), 7.47 (s, 1H), 7.34 (d, *J* = 7.6 Hz, 1H), 6.56 (d, *J* = 2.3 Hz, 1H), 6.52–6.35 (m, 2H), 3.36 (q, *J* = 7.0 Hz, 4H), 2.80 (d, *J* = 15.6 Hz, 1H), 2.67 (s, 1H), 1.91 (d, *J* = 12.9 Hz, 1H), 1.62 (d, *J* = 9.5 Hz, 3H), 1.11 (t, *J* = 6.9 Hz, 6H). ^13^C NMR (75 MHz, DMSO-*d*_6_) *δ* 191.99, 149.12, 142.35, 134.78, 134.40, 132.23, 129.65, 129.53, 129.04, 127.80, 124.44, 123.99, 123.39, 109.19, 96.69, 43.43, 26.41, 22.36, 21.54, 12.05. ESI-MS calcd for C_32_H_30_NO_4_ [M + H]^+^ 492.2133, found 492.2160.

### 2.3. Preparation of reactive oxygen species (ROS) and reactive nitrogen species (RNS), active sulfur species (RSS) and anions

H_2_O_2_ was prepared by the direct dilution of a commercial hydrogen peroxide stock solution. NaClO was obtained by the dilution of a commercial hypochlorite solution in purified water and measured using a spectrophotometer (*ε*_292 nm_ = 350 M^−1^ cm^−1^). TBHP was prepared by the dilution of a commercial *tert*-butyl hydroperoxide stock solution. Peroxynitrite stock (ONOO^−^) was prepared by a previously reported procedure^23^ and measured using a spectrophotometer (*ε*_302 nm_ = 1670 M^−1^ cm^−1^) 1 M of stock solutions (Cys, GSH, HS^−^, SO_4_^2−^, S_2_O_3_^2−^, S^2−^, SCN^−^, H_2_O_2_, ClO^−^, TBHP, Hcy, NO_2_^−^, Sx^2−^, Cl^−^, Br^−^, I^−^, CO_3_^2−^, HCO_3_^−^, PO_4_^2−^, HPO_4_^−^, AcO^−^, SO_3_^2−^, and HSO_3_^−^) were prepared by the dissolution of 10 mmol solid in purified water, and diluted to the desired concentrations when needed.

### 2.4. pH value adjustment

The pH values of the solutions were directly obtained by preparing a series of specific pH buffers, including acetate buffer, phosphate buffer, and sodium hydroxide/potassium chloride/boric acid buffer.

### 2.5. Spectral analysis

1 mM of the probe stock solution was prepared by dissolving 1 mg Mito-CDTH-CHO in 2 ml anhydrous ethanol, and diluted with PBS solution (10 mM, pH = 7.4, 6.0 or 8.0, containing 2% EtOH) for final test solutions.

### 2.6. Cell cytotoxicity assay

The cytotoxicity was measured using a CCK-8 kit. Hela cells were cultured in Dulbecco's modified Eagle's medium (DMEM) containing 10% fetal bovine serum (FBS) and 1% antibiotics at 37 °C under 5% CO_2_ for 24 h. Hela cells were cultured with a fresh medium containing various concentrations of Mito-CDTH-CHO (0–40 μM) for another 12 h. Next, Hela cells were washed three times with PBS and incubated with diluted CCK-8 reagent for 1 h, and then the cell viability was determined by a microplate reader. The procedure was repeated three times for each concentration.

### 2.7. Cell culture and imaging

HepG-2 cells were cultured in a DMEM medium (containing 1% penicillin/streptomycin and 10% FBS) under an air condition at 37 °C under 5% CO_2_. HepG-2 cells at the logarithmic growth phase were implanted into 25 mm glass-bottomed dishes and incubated overnight. After the attachment of cells, the cells were treated with different pH values (pH = 6.0, 7.4 and 8.0) of the DMEM medium for 3 h. The pH of the DMEM medium was adjusted by adding a specific concentration of hydrochloric acid or sodium hydroxide.^23^ HepG-2 cells were washed with PBS three times and incubated with Mito-CDTH-CHO (20 μM) in an untreated DMEM medium. Confocal fluorescence images were recorded using a Zeiss LSM 800 confocal laser scanning microscope. The green channel was collected at 460–520 nm at an excitation of 390, and the blue fluorescence channel was covered over the range of 420–470 nm at an excitation of 370 nm.

## 3. Results and discussions

### 3.1. Design and synthesis of Mito-CDTH-CHO

**Scheme 1:**
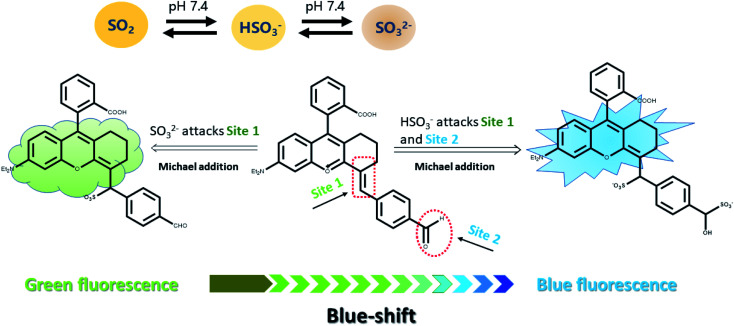
Rational design and sensing mechanism of the probe for HSO_3_^−^ and SO_3_^2−^.

According to the previous reports on the response mechanism of detecting sulfur dioxide type fluorescent probes,^24–30^ we proposed that (Scheme 1), on the one hand, the oxygen positive ion on the benzopyran ring acts as a strong electron-withdrawing group, which reduces the electron cloud density of the C=C double bond and enable the C=C double bond strong electrophilicity. On the other hand, the C=O double bond conjugated to the benzene ring also possesses weaker electrophilicity, which makes the strong nucleophilic HSO_3_^−^ attack the C=O and C=C double bonds. When the probe Mito-CDTH-CHO conjugate structure is broken, strong blue fluorescence is emitted. The weaker nucleophilic SO_3_^2−^ could only attack the C=C double bond, hence exhibited red-shifted green fluorescence.

### 3.2. Vitro sensing of Mito-CDTH-CHO for SO_3_^2−^/HSO_3_^−^

**Fig. 1:**
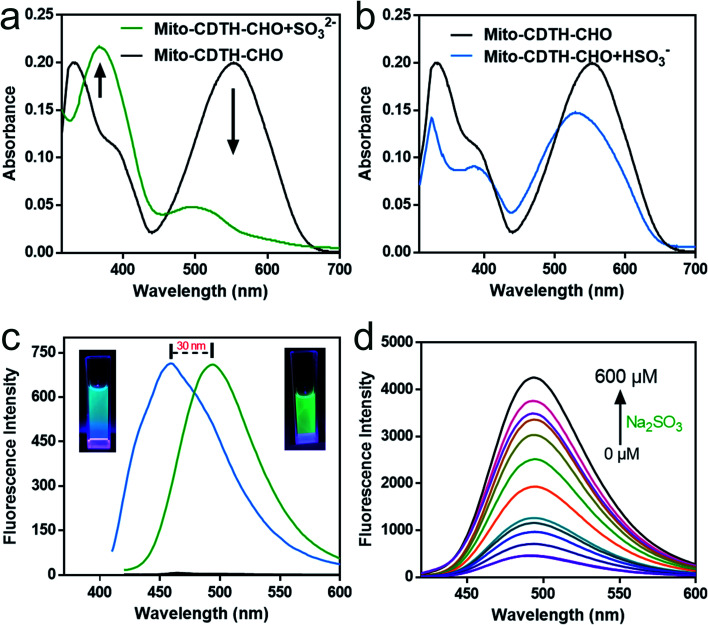
Fluorescence and UV responses of Mito-CDTH-CHO toward SO_2_ derivatives. (a) The UV-Vis absorption spectra change of Mito-CDTH-CHO (20 μM) with Na_2_SO_3_ (20 μM) in PBS buffer (pH 7.4, containing 2% EtOH). (b) UV-Vis absorption spectra change of Mito-CDTH-CHO (20 μM) with NaHSO_3_ (20 μM) in PBS buffer (pH 6.0, containing 2% EtOH). (c) Fluorescence spectra changes of Mito-CDTH-CHO (20 μM) with 50 μM SO_2_ derivatives in PBS buffer (containing 2% EtOH). Black: Mito-CDTH-CHO; blue: NaHSO_3_, *λ*_ex_ = 370 nm; green: Na_2_SO_3_, *λ*_ex_ = 390 nm. (d) Fluorescence spectra changes of Mito-CDTH-CHO (20 μM) in the presence of various concentrations Na_2_SO_3_ (0–600 μM), *λ*_ex_ = 390 nm.

First of all, we tested the UV-Vis absorption of Mito-CDTH-CHO in the absence and presence of SO_2_ derivatives (SO_3_^2−^ and HSO_3_^−^) in PBS buffer. There were mainly two absorption peaks at 300–700 nm, centered at 330 nm and 553 nm, respectively. Mito-CDTH-CHO exhibited similar UV spectra changes after it reacted with SO_3_^2−^ (Fig. 1a) and HSO_3_^−^ (Fig. 1b). The absorption intensity at 553 nm sharply decreased, which means for the breaking of the conjugate system, and a new absorption at 365 nm was elevated.

To explain the fluorescence response distinction, the dual-site fluorescence response of probe toward SO_3_^2−^ and HSO_3_^−^ was investigated. As shown in Fig. 1c, the probe Mito-CDTH-CHO (20 μM) had almost no fluorescence in the absence of SO_3_^2−^/HSO_3_^−^ at 400–600 nm. However, after the reaction with 50 of μM Na_2_SO_3_, the strong green fluorescence was emitted (*λ*_ex_ = 390 nm, *λ*_em_ = 492 nm). When at the same concentration in the case of NaHSO_3_, the solution exhibited luminous blue emission at a shorter wavelength (*λ*_ex_ = 370 nm, *λ*_em_ = 456 nm). These results provide a preliminary proof that the probe Mito-CDTH-CHO conjugate structure is destroyed by SO_3_^2−^ and HSO_3_^−^.

**Fig. 2:**
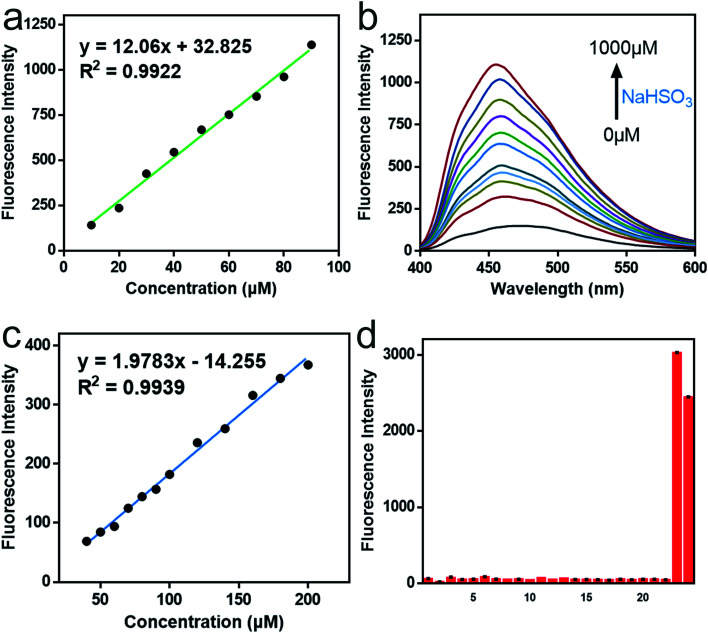
Fluorescence responses of Mito-CDTH-CHO toward SO_2_ derivatives. (a) The linear curve of Mito-CDTH-CHO (20 μM) fluorescence intensity at 492 nm with Na_2_SO_3_ concentrations range from 10–100 μM. (b) Fluorescence spectra changes of Mito-CDTH-CHO (20 μM) after the addition of various concentrations NaHSO_3_ (0–1000 μM). PBS buffer, pH 6.0, containing 2% EtOH, *λ*_ex_ = 370 nm. (c) The linear curve of Mito-CDTH-CHO (20 μM) fluorescence intensity at 456 nm with NaHSO_3_ concentrations range from 40–200 μM in PBS buffer. (d) The fluorescence intensity values of Mito-CDTH-CHO (20 μM) after interacting with 500 μM SO_2_ derivatives, reactive nitrogen species, reactive oxygen species, active sulfur species and anions. (1) Mito-CDTH-CHO, (2) Cys, (3) GSH, (4) HS^−^, (5) SO_4_^2−^, (6) S_2_O_3_^2−^, (7) S^2−^, (8) SCN^−^, (9) H_2_O_2_, (10) NaClO, (11) TBHP, (12) Hcy, (13) NO_2_^−^, (14) Sx^2−^, (15) Cl^−^, (16) Br^−^, (17) I^−^, (18) CO_3_^2−^, (19) HCO_3_^−^, (20) PO_4_^2−^, (21) HPO_4_^−^, (22) AcO^−^, (23) SO_3_^2−^, (24) HSO_3_^−^.

As shown in Fig. 1d and 2a, for SO_3_^2−^, with the addition of the Na_2_SO_3_ (0–600 μM), the emission intensity at 492 nm increased significantly (pH = 7.4, 37 °C, *λ*_ex_ = 390 nm), and an excellent linear relationship (*R*^2^ = 0.992) was obtained in the range of 10–100 μM. Moreover, the detection limit was calculated to be 100 nM. For HSO_3_^−^, Fig. 2b and c revealed that the fluorescence intensity at 456 nm constantly increased after the addition of 0–1000 μM NaHSO_3_ in the phosphate buffer solution (pH = 6.0, 37 °C, *λ*_ex_ = 370 nm), in a wide linear range (40–200 μM). The detection limit was 80 nM (Fig. 2c).

Afterward, we evaluated the selectivity and pH stability of the probe Mito-CDTH-CHO towards sulfur dioxide derivatives. As described in Fig. 2d, SO_3_^2−^/HSO_3_^−^ led to a significant fluorescence enhancement at 492 nm and 456 nm, respectively. While other interfering substances, including reactive nitrogen species (NO_2_^−^ and NO_3_^−^), reactive oxygen species (ClO^−^, H_2_O_2_, TBHP), active sulfur species (SO_4_^2−^, HS^−^, S_2_O_3_^2−^, S^2−^, SCN^−^, Sx^2−^, Cys, Hcy, GSH) and anions (I^−^, Br^−^, Cl^−^, CO_3_^2−^, HCO_3_^−^, PO_4_^3−^, HPO_4_^2−^, ACO^−^), did not produce remarkable fluorescence response. From what had been discussed above, Mito-CDTH-CHO presented excellent selectivity toward SO_3_^2−^ and HSO_3_^−^ in separated emission regions (492 and 456 nm).

The pH interference of the probe for SO_2_ derivatives was discussed. In the absence of SO_3_^2−^ and HSO_3_^−^, the probe had little fluorescence and was unaffected by the variation of pH values (Fig. S5†). When Na_2_SO_3_ or NaHSO_3_ was added, the fluorescence intensity changed with the mutual conversion balance between SO_3_^2−^ and HSO_3_^−^ in the range of pH 4 to 10 (Fig. S6†). In the range of acidic pH (4–6), HSO_3_^−^ ion dominates, thus Mito-CDTH-CHO exhibited stronger fluorescence at 456 nm than in neutral and weak basic pH ranges (Fig. S7†). In basic pH ranges (7–10), SO_3_^2−^ accounts for the main part, so the fluorescence intensity increased with the pH value increase (Fig. S6†).

In general, compared to other fluorescence probes sensing SO_2_, the most evident superiority of Mito-CDTH-CHO is selectivity for SO_3_^2−^ and HSO_3_^−^. Most fluorescent probes for detecting SO_2_ are not selective toward SO_3_^2−^ and HSO_3_^−^ due to their very similar chemical properties, showing that the same response toward SO_3_^2−^ (HSO_3_^−^) when detecting HSO_3_^−^ (SO_3_^2−^). In addition, superior water solubility, suitable detection limit, and accurate mitochondrial targeting performance also indicate that Mito-CDTH-CHO is a fairly qualified fluorescent probe for the accurate detection of sulfur dioxide derivatives (see Table S1†).

### 3.3. The proposed mechanism of Mito-CDTH-CHO for SO_2_ derivatives detection

**Fig. 3:**
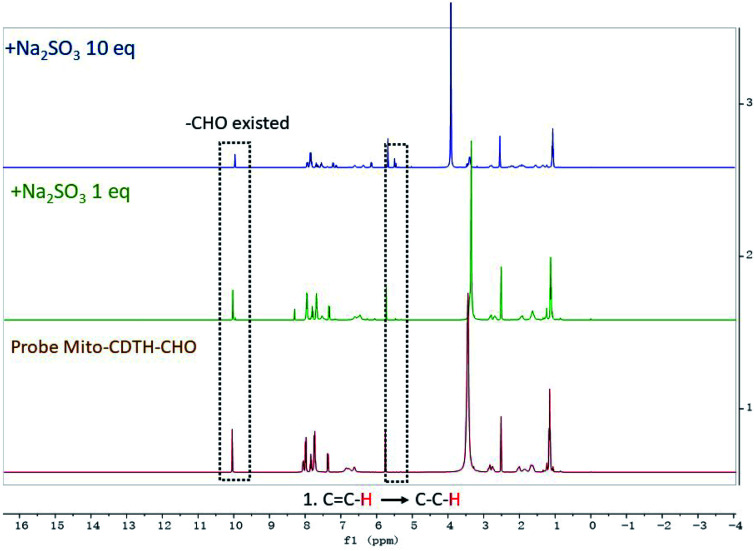
The ^1^H NMR titration of Mito-CDTH-CHO with Na_2_SO_3_.

**Fig. 4:**
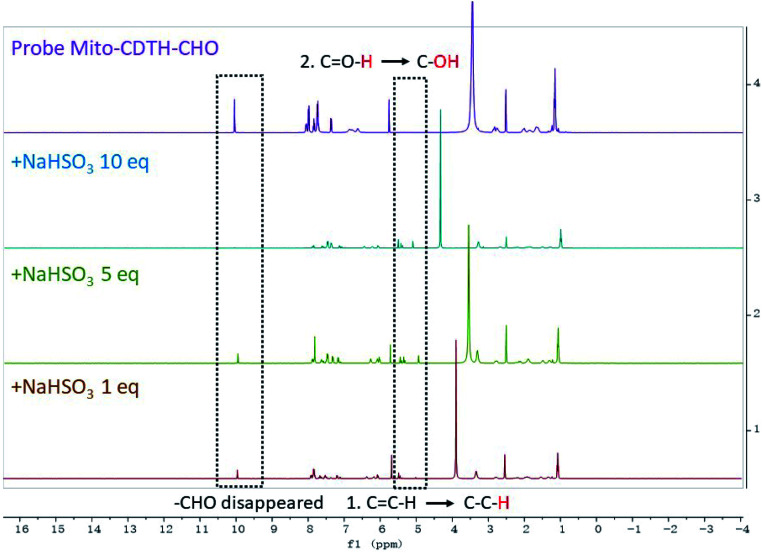
The ^1^H NMR titration of Mito-CDTH-CHO with NaHSO_3_.

In order to illuminate the reaction mechanism between Mito-CDTH-CHO and SO_2_ derivatives, the NMR titration experiments in DMSO-*d*_6_/D_2_O (8 : 2) were performed. As shown in Fig. 3, a proton at 6.34 ppm represents the double bond conjugated to benzopyrone of the probe Mito-CDTH-CHO. As the concentration of Na_2_SO_3_ increased from 1 to 10 eq., the proton peak disappeared, while the singlet at 4.89 ppm appeared. The response mechanism of the probe Mito-CDTH-CHO to NaHSO_3_ was also confirmed (Fig. 4), except the singlet at 4.89 ppm, an additional hydroxyl proton peak at 5.12 ppm emerged, which is attributed to the difference in nucleophilicity between SO_3_^2−^ and HSO_3_^−^. These results further verified that the dual-site sensing of Mito-CDTH-CHO toward Na_2_SO_3_ and NaHSO_3_*via* different double bond nucleophilic addition reactions.

### 3.4. Cellular imaging of Mito-CDTH-CHO

**Fig. 5:**
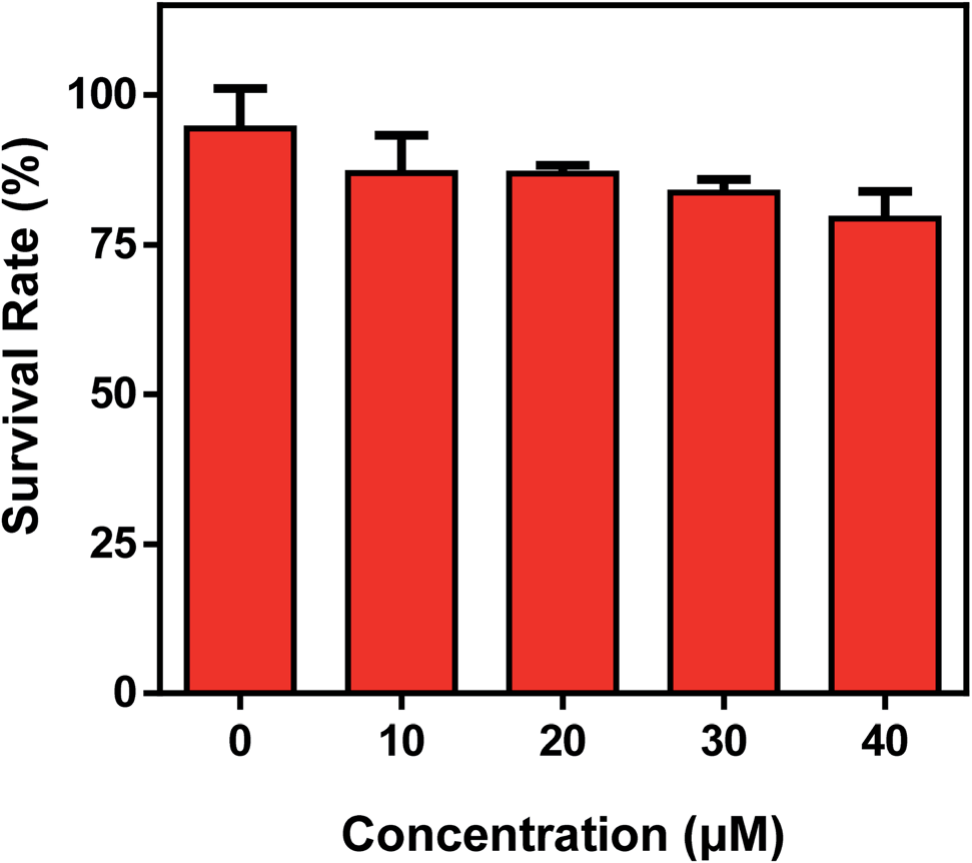
Cell viability of Mito-CDTH-CHO in a standard CCK-8 kit in living HepG-2 cells for 24 h. The experiment was repeated three times (±S.D.).

In view of the excellent performances of the probe Mito-CDTH-CHO*in vitro*, the capability of the discriminative detection of SO_3_^2−^ and HSO_3_^−^ was investigated. Prior to bioimaging experiments, the cytotoxic assay was carried out by a CCK-8 method in HepG-2 cells, the results indicated that Mito-CDTH-CHO had low cytotoxicity (Fig. 5).

**Fig. 6:**
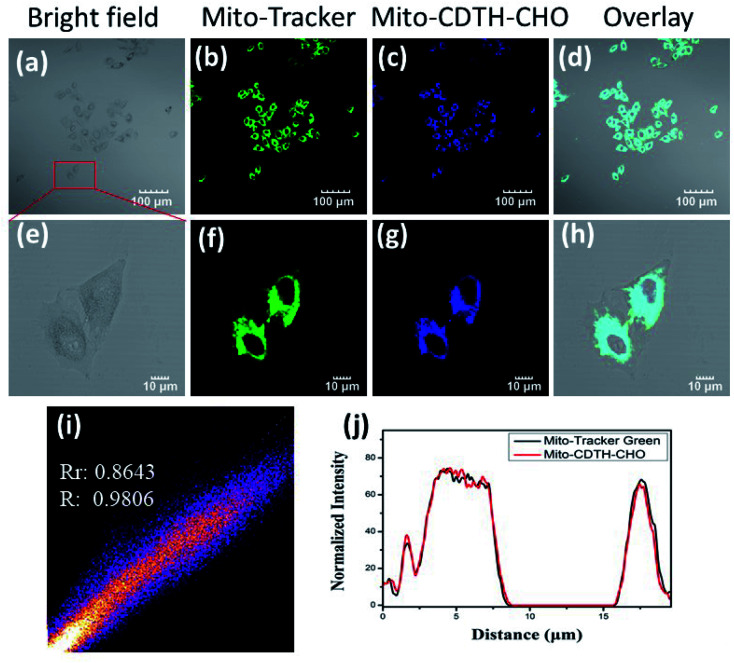
Confocal microscopy colocalization images of Mito-CDTH-CHO and Mito-Tracker Green in HepG-2 cells. (a and e) the bright field of HepG-2 cells. (b and f) fluorescence image of Mito-Tracker Green (*λ*_ex_ = 488 nm, *λ*_em_ = 500–540 nm). (c and g) fluorescence image of Mito-CDTH-CHO (*λ*_ex_ = 370 nm, *λ*_em_ = 400–460 nm). (d and h) merged image of (b and c), (f and g), respectively. (i) Intensity scatter plot of blue and green channels and (j) normalized intensity profile of the linear region of part a across the HepG-2 cells.

According to the literature,^31–33^ cationic small molecules could enter into mitochondria and interact with anionic species *via* the electrostatic interaction. The design of Mito-CDTH-CHO is based on our considerations that the benzopyran cation (containing oxygen positive ions) can act as a mitochondrion-targeting moiety. The positive charge and hydrophobic properties of the benzopyran cation are supposed to mediate the localization of Mito-CDTH-CHO inside the mitochondrial membrane. Thus, we speculate that Mito-CDTH-CHO will mainly distribute in the mitochondria. In order to verify our hypothesis, the mitochondrial colocalization experiment was carried out. The commercial mitochondrion tracker (Mito-tracker) and Mito-CDTH-CHO were co-incubated in HepG-2 cells. The fluorescence imaging from Mito-CDTH-CHO in the blue channel (Fig. 6c and g) overlapped well with the Mito-tracker in the green channel (Fig. 6b and f), resulting in the Pearson's correlation coefficient of 0.98. Furthermore, the region of interest (ROI) is illustrated in Fig. 6j, and the normalized fluorescence intensity of Mito-CDTH-CHO changed in coordination with the normalized fluorescence intensity of Mito-Tracker Green. These results suggested that Mito-CDTH-CHO possesses the excellent ability to target mitochondrial of subcellular organelle in HepG-2 cells.

**Fig. 7:**
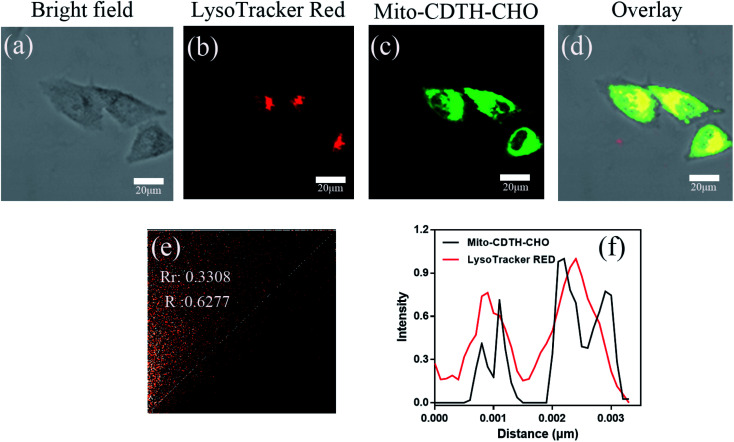
Confocal microscopy colocalization images of Mito-CDTH-CHO and Mito-Tracker RED in HepG-2 cells. (a) Bright field of HepG-2 cells. (b) Fluorescence image of Mito-Tracker RED (*λ*_ex_ = 548 nm, *λ*_em_ = 560–620 nm). (c) Fluorescence image of Mito-CDTH-CHO (*λ*_ex_ = 390 nm, *λ*_em_ = 460–520 nm). (d) The merges images of (a and b). (e) Intensity scatter plot of red and green channels. (f) Normalized intensity profile of the linear region of part a across the HepG-2 cells. Scale bar = 20 μm.

For the sake of better experimental results, we conducted the control experiments with a lyso-tracker. As illustrated in Fig. 7, the green fluorescence of Mito-CDTH-CHO is not overlapped at all with the red fluorescence of LysoTracker RED with the Pearson's correlation coefficients (*R*_r_) of 0.3308 and an overlap coefficient (*R*) of 0.6277 (Fig. 7e). The green fluorescence of Mito-CDTH-CHO and red fluorescence of LysoTracker RED changes in the intensity profiles of ROIs are not synchronized at all (Fig. 7f). The result further indicates that Mito-CDTH-CHO mainly localizes in the mitochondria of living cells.

**Fig. 8:**
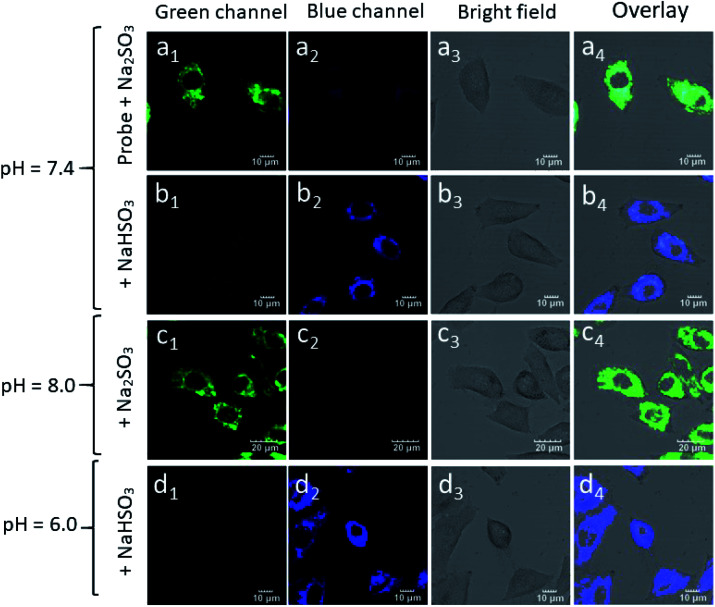
The confocal fluorescence images of probe and SO_2_ derivatives in HepG-2 cells. (a_1_–a_4_) HepG-2 cells were pre-treated with Mito-CDTH-CHO (20 μM), then incubated with Na_2_SO_3_ (200 μM) for 30 min. (b_1_–b_4_) HepG-2 cells were incubated with NaHSO_3_ (200 μM) for 30 min. (c_1_–c_4_) After incubated with Na_2_SO_3_ (200 μM) for 30 min. (d_1_–d_4_) After incubated with NaHSO_3_ (200 μM) for 30 min. All cells were pre-treated with different pH values (pH = 6.0, 7.4 and 8.0) DMEM medium for 3 h.

HepG-2 cells were pre-treated with a probe (20 μM) in the DMEM medium and then incubated with Na_2_SO_3_ (200 μM) for 30 min. As shown in Fig. 8, due to the equilibrium conversion between SO_3_^2−^ and HSO_3_^−^ in a neutral fluid, HepG-2 cells exhibited distinct fluorescence (a_1_) in the green channel and weak fluorescence (a_2_) in the blue channel. In contrast, after incubating the HepG-2 cells with NaHSO_3_ (200 μM) for 30 min, clear fluorescence in the blue channel (b_1_) and weak fluorescence (b_2_) in the green channel were observed. Inspired by the above experimental results, HepG-2 cells were cultured with NaHSO_3_ (200 μM) for 30 min, it is worth noting that the fluorescence in the green channel disappeared (d_1_), whereas the fluorescence in the blue channel enhanced (d_2_). Similarly, in the HepG-2 cells incubated with Na_2_SO_3_ in the DMEM medium for 30 min, there was fluorescence enhancement in the green channel (c_1_) and almost no fluorescence in the blue channel (c_2_). Therefore, probe Mito-CDTH-CHO can detect the intracellular SO_3_^2−^ and HSO_3_^−^ levels with different fluorescence signals.

## 4. Conclusions

In short, to the best of our knowledge, for the first time, a dual-site fluorescence probe for HSO_3_^−^ and SO_3_^2−^ with two different emission signals was designed and synthesized. Mito-CDTH-CHO can distinguishingly sense the levels of HSO_3_^−^ and SO_3_^2−^ with different fluorescence signals under separate pH conditions in living biological systems and possesses low cytotoxicity, excellent biocompatibility and outstanding mitochondrial targeting. In the meantime, the sensing mechanism of the double bond nucleophilic addition was successfully validated using the NMR titration experiments. We envision that Mito-CDTH-CHO could provide a deeper insight into and a better understanding of the physiological and pathological processes of SO_2_ derivatives.

## Conflicts of interest

There are no conflicts to declare.

## Supplementary Material

RA-010-D0RA01233E-s001
