# Supplementary material for: A mitochondrion-targeted dual-site fluorescent probe for the discriminative detection of SO32− and HSO3− in living HepG-2 cells
Source: RSC Adv. 2020 Jul 14;10(44):26349–57. doi: 10.1039/d0ra01233e (PMC9055423; doi:10.1039/d0ra01233e)
Supplement: RA-010-D0RA01233E-s001 [file RA-010-D0RA01233E-s001.pdf]

Electronic Supplementary Material (ESI) for RSC Advances.
This journal is © The Royal Society of Chemistry 2020

Detection limit

The detection limit is calculated by Equation 1, as shown below:

$$Detection\ limit = 3\sigma/\kappa \qquad (1)$$

In which σ is the standard deviation of the blank measurements, by measuring the probe (20μM) of emission intensity without Na_2SO_3 and $NaHSO_3$ for ten times. κ is the slope of the intensity *vs* concentrations of Na_2SO_3 or $NaHSO_3$.

Scheme S1. Synthesis route of probe **Mito-CDTH-CHO**

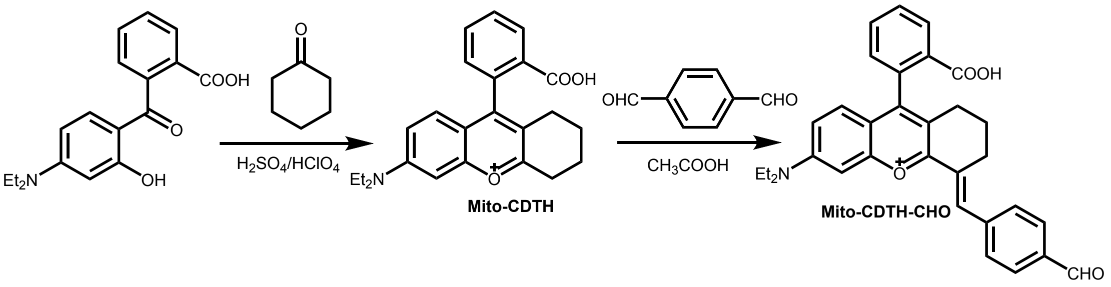

Characterization data for synthesis

Fig S1: ^{1}H-NMR spectrum of probe **Mito-CDTH-CHO** in DMSO-*d6*

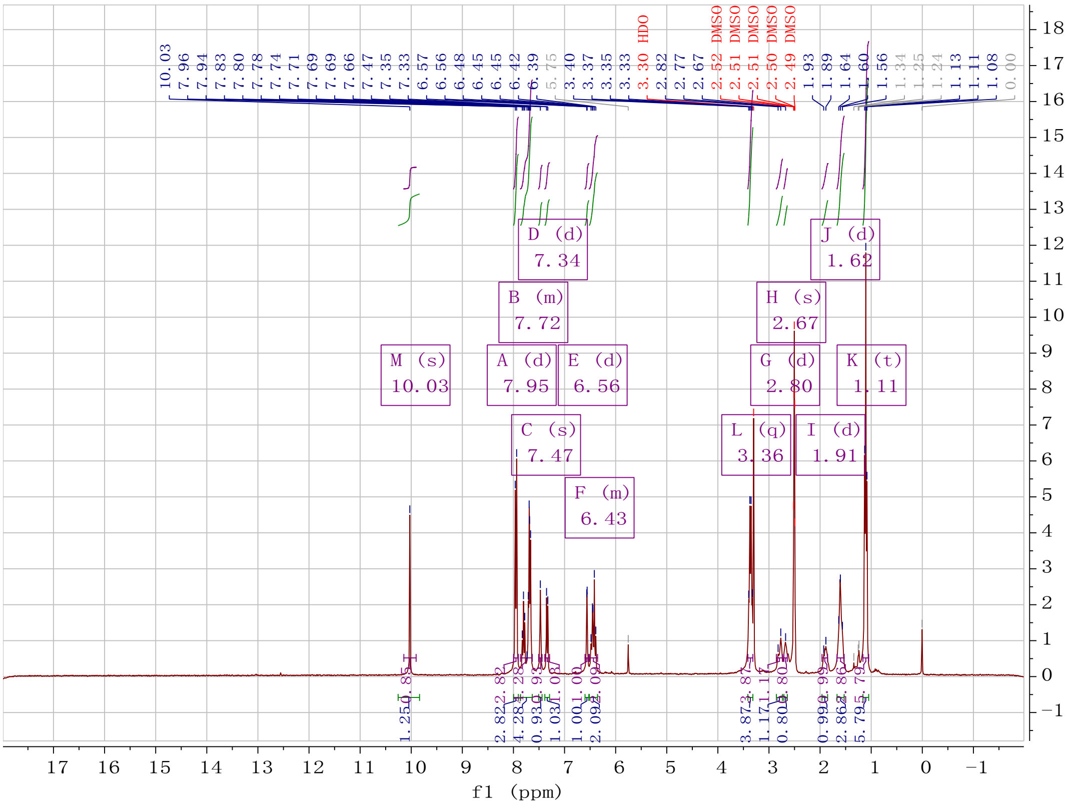

Fig S2: ^{13}C-NMR spectrum of probe **Mito-CDTH-CHO** in DMSO-*d6*

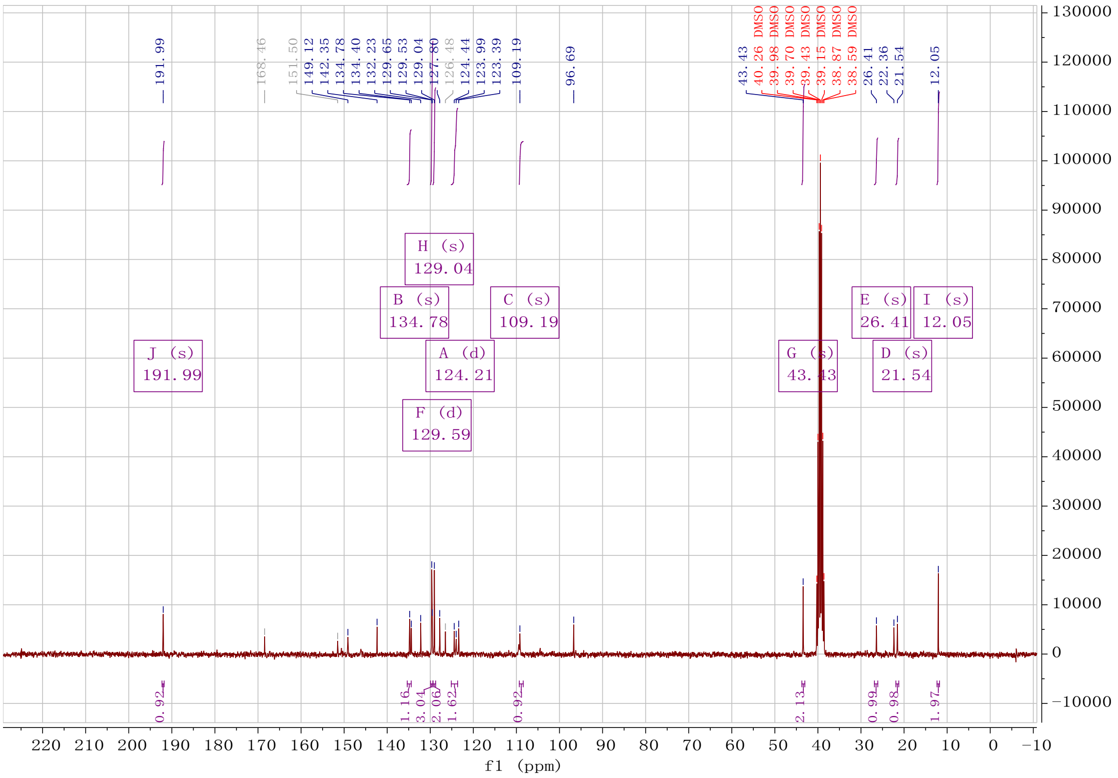

Fig S3: ESI-MS of **Mito-CDTH**

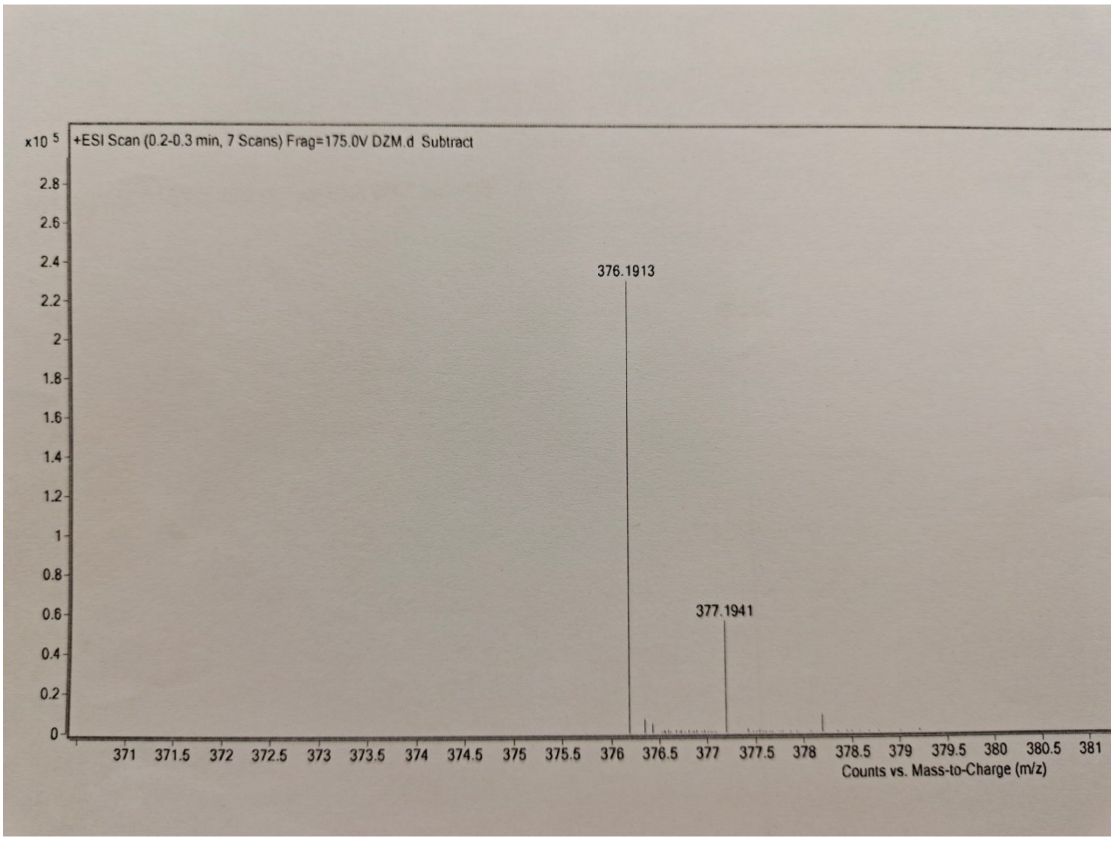

Fig S4: ESI-MS of **Mito-CDTH-CHO**

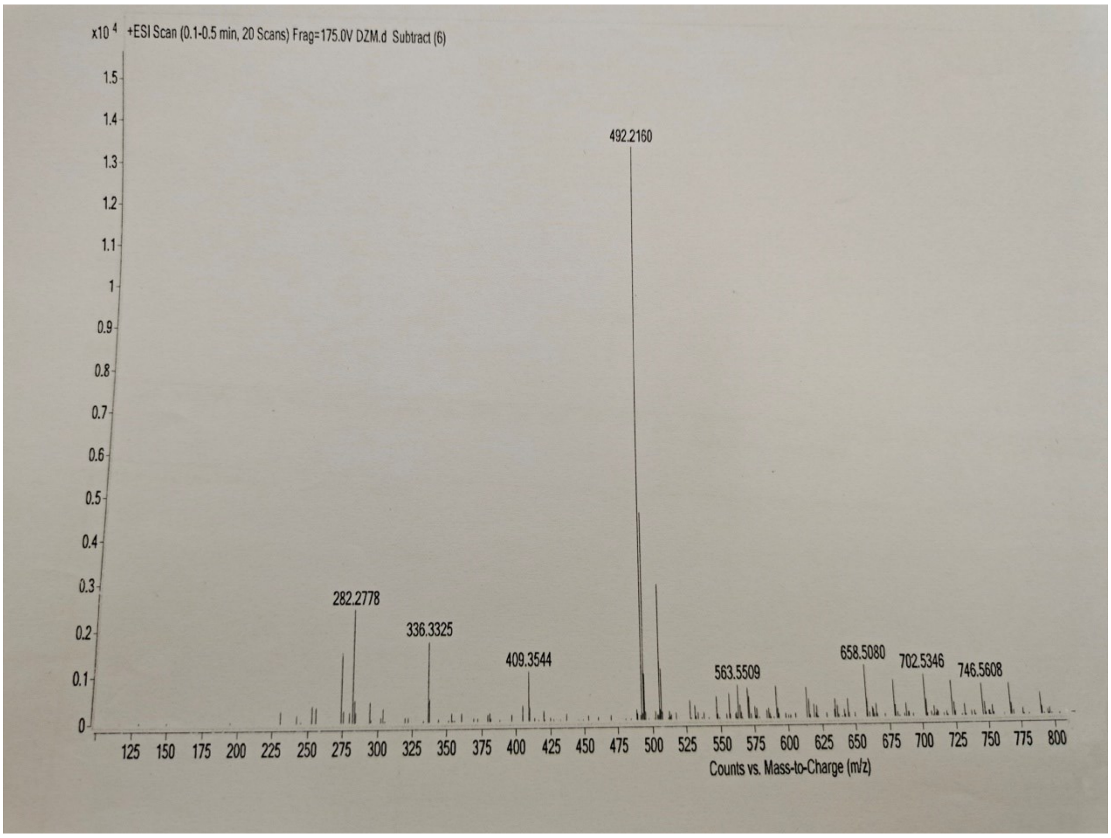

Fig S5: Fluorescence spectra changes of **Mito-CDTH-CHO** (20μM) with 50μM SO_2 derivatives in PBS buffer (containing 1% DMSO). Red: **Mito-CDTH-CHO**; Blue: $NaHSO_3$, λex = 370 nm; Green: Na_2SO_3, λex = 390 nm.

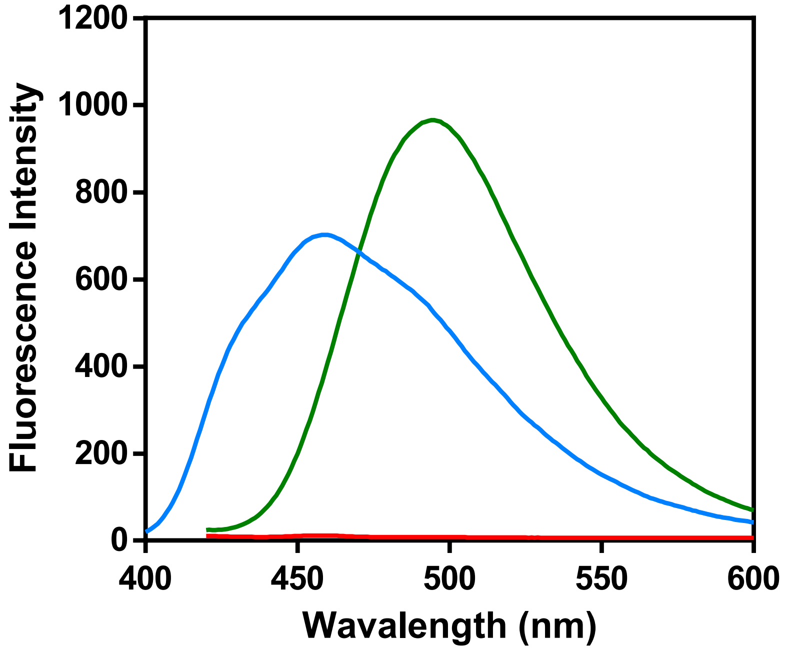

Fig S6: The fluorescence intensity of **Mito-CDTH-CHO** with Na_2SO_3 with diverse pH values.

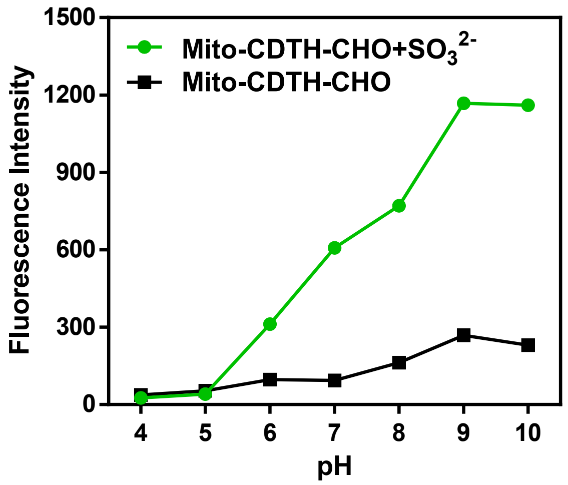

Fig S7: The fluorescence intensity of **Mito-CDTH-CHO** with $NaHSO_3$ with diverse pH values.

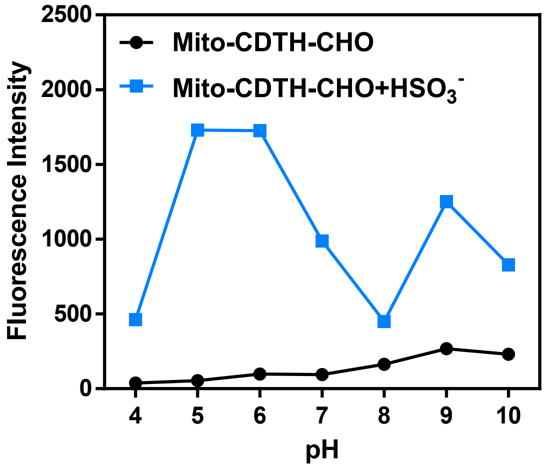

1

Table S1: A comparison of mitochondria-targeted dual-site fluorescent probes for SO_3^{2-} and HSO_3^-

Probe	Dual-site	water solubility	LOD	Response time	Targeting mitochondria	Selectivity for SO_3^{2-} and HSO_3^-
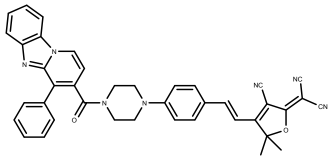 RCS ADV. 2019, 9,1147	No	DMF/PBS 3:7	62nM	2 min	No	No
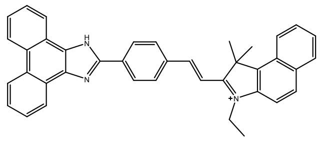 Talanta. 2019, 191, 428-434	No	EtOH/PBS 4:6	26 nM	30 min	Yes	No
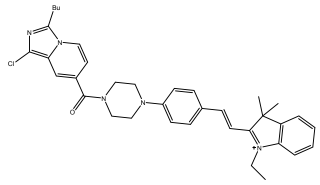 RCS ADV. 2019, 9, 8943	No	PBS	130nM	3 min	Yes	No
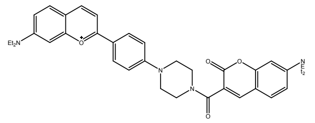 SENSOR ACTUAT B-CHEM. 2019, 284, 330-336	No	DMF/H_2O 1:9	17.7nM	60 s	Yes	No
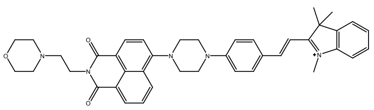 SENSOR ACTUAT B-CHEM. 2019, 292,	No	DMSO/PBS	820nM	30 min	Yes	No

80-87						
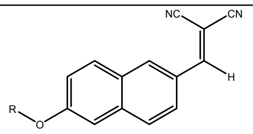<br>J MATER CHEM B. 2013, 00, 1-3	No	DMSO/PBS 1:9	15.5nM	50 s	Yes	No
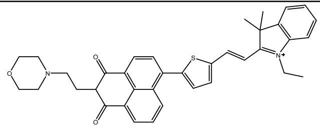<br>Anal. Chem. 2019, 91, 11946-11951	No	5% DMSO in PBS	20.7nM	200 s	No	No
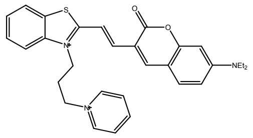<br>NEW J CHEM. 2012, 00, 1-3	No	DMSO/PBS	29.2μM	1 min	Yes	No
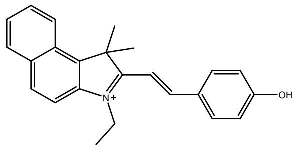<br>Molecules. 2019, 24, 4011	No	DMSO/PBS	28nM	30 min	No	No
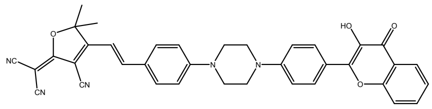<br>Dyes Pigm. 2018, 151, 95-101	No	EtOH/PBS 4:6	17nM	30 min	No	No
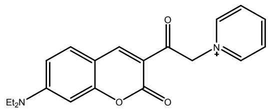<br>SENSOR ACTUAT B-CHEM. 2019, 295, 215-222	No	THF/PBS 3:1	Not mentioned	15 min	Yes	No
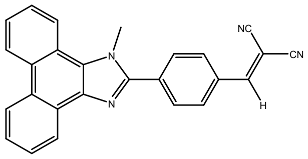<br>Biomaterials. 2017, 183, 82-93	No	DMSO/PBS 50:50	Not mentioned	60 s	No	No
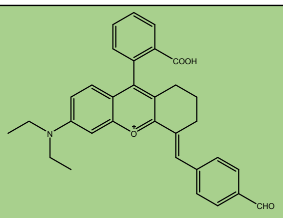<br>This work	Yes	2% EtOH in PBS	100 and 80nM	Within 10 min	Yes	Yes

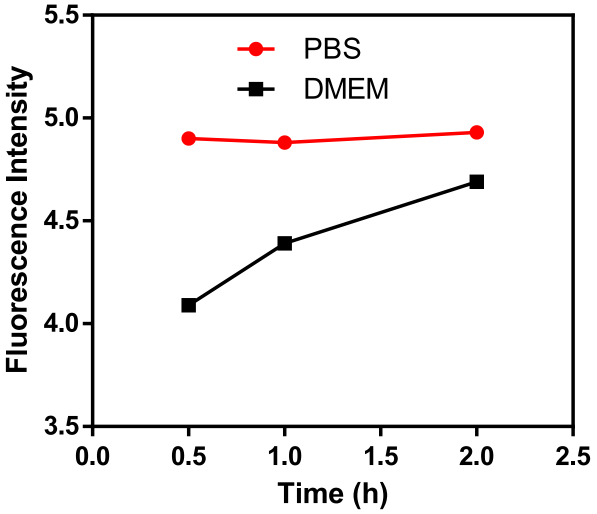

Fig. S8: The fluorescence intensity changes of Mito-CDTH-CHO without SO_2 derivatives in PBS and DMEM medium within 2 h
